# The Causes of Hemorrhage from the Unimpregnated Uterus

**Published:** 1855-11

**Authors:** M. M. Pallen

**Affiliations:** Professor of Obstetrics, &c., in the St. Louis Medical College


### SELECTIONS.

*The Causes of Hemorrhage from the Unimpregnated Uterus.* By M. M. Pallen, M. D., Professor of Obstetrics, &c., in the St. Louis Medical College.

I commence this paper by stating, that I do not expect to offer any thing new. I write it, because in the commencement of my medical career, I was too apt to overlook the causes of uterine hemorrhage, and because I fear that there are others who may fall into the same error. So much is said in the medical books, concerning idiopathic hemorrhage, either active or passive, that young practitioners when called to such cases, naturally enough, frequently neglect to ascertain the causes ot the flow.

I do not pretend to assert that idiopathic hemorrhage from the uterus does never exist, but I affirm that it is not the condition, in the majority of cases, and I believe that it is so only in a very small proportion. I believe that in a large per centage of cases, there is a local cause for the bleeding, and it is the duty of the physician, if possible, to ascertain such cause.

Perhaps it would not be incorrect to assert, that there is really no idiopathic hemorrhage from the uterus—an active idiopathic hemorrhage is but an aggravation of the menstrual discharge. There is an active congestion on the organ—something more than the physiological congestion attendant on the menstrual flow—a condition akin to active inflammation—and the flow is the means nature adopts for the relief of the organ. The cause is the active congestion.

If the flow continues too long, the patient becomes anemic; or, in other words, the blood becomes too thin, and the uterine vascular tissue relaxed, and the result is a passive hemorrhage. Or the same condition of the fluids and solids may be produced by other exhausting discharges, and passive hemorrhage is the consequence. Here the cause is in the blood itself, and in the relaxed solids. But leaving the dispute about words out of the question, the facts are such, as I have represented them; and such conditions producing hemorrhages are called idiopathic hemorrhages. They occur, however, as I have stated, not near so often, as other causes which produce similar results.—Let us then consider these causes. Hemorrhage from the unimpregnated uterus is sometimes the result of chronic inflammation of the lining membrane of that organ. Such a hemorrhage may commence at the usual menstrual period and continue for some days, or a week, or two weeks, or longer, beyond the usual period of menstruation. Then it will disappear and commence again at the next menstrual term. The intervals of non-appearance become less and less in duration, as time advance, until they totally vanish. The female loses blood all the time.

Sometimes the flow ceases, and commences at any uncertain time, so that the patient has no regular periods at all.

Another cause of uterine hemorrhage is, inflammation and ulceration of the neck of the womb. This has been repeatedly denied. Dr. West in his lectures has endeavored to prove that such lesions of the uterus have had an undue importance attached to them. To such a general proposition I assent. It is true of this local affection, as it is true of many other local affections. Stricture of the urethra is a grave malady—produces in very many cases much suffering and bad health. Yet I have never seen men in bad health, with slight stricture, who have been treated as if the stricture were the sole cause of the derangement of the system—whereas, if the stricture alone existed, the patients would get along with tolerable comfort, but unfortunately there were other sadder conditions over which bougies, or the knife, possessed no control whatsoever.

So it is with slight abrasions on the neck of the womb. They are but an unimportant part of the general ill health; and their cure will not do much to restore health to the patient.

But on the other hand, such lesions do produce serious disturbances, and among these are hemorrhages. I do not mean a slight inflammation and a slighter abrasion—I mean extensive disorganization—as when the epithelium is peeled off—the villi enlarged; so as to appear as if there were extensive granulations, the neck enlarged, and above all the os uteri patulent, and the lips exerted. The ulceration (I use the term for want of a better one) can then be perceived to extend into the neck of the womb. I have seen such cases attended with hemorrhage, (some of them of protracted date,) and I have found the hemorrhage to cease, when the neck of the womb was cured. This is, for me, sufficient evidence of the cause of the flow, notwithstanding the ingenious book of Dr. West.

Hemorrhage is sometimes the result of an enlargement of the neck and body of the uterus. These organs have been the seat of chronic inflammation, and examination detects an enlargement. I once had a patient, who had been the subject of hemorrhage for several months. She complained of the usual symptoms of uterine disease. I found the neck of the womb very much enlarged and somewhat indurated. She was directed to keep quiet, and I applied daily compound iodine ointment to the neck. In the course of three months, she was entirely restored to health. Of this mode of treatment I intend to say more in connection with tumors of the womb. At present, it is sufficient to refer to the above cause of hemorrhage.

Polypi of the womb have been long known as causes of hemorrhage—when the polypus is at the os uteri, or when it grows internally, and passes out of the os, it is early detected. But I fear that it occasionally happens, that an internal polypus, or a sub-mucous tumor exists, produces an exhausting hemorrhage, and is never suspected.

A lady told me that her sister died from a hemorrgage, which lasted a long time,—the cause of which was never ascertained during life. Examination after death revealed a small tumor projecting into the cavity of the womb.

The important question then arises, how are we to discover the existence of such tumors? I confess that it is sometimes very difficult. If the tumor be very large, examination through the rectum and the vagina may be sufficient. But if such be not the case, then the diagnosis is obscure. If we cannot ascribe the hemorrhage to any other cause, then the supposition might be very strong, that it arose from a tumor. The frequency of such growths would lead to such a surmise.—Bayle has stated, and Dr. Lee agrees with him, that they exist in twenty out of a hundred middle-aged women.

It has recently been proposed by Dr. Barnes, of London, to dilate the os and cervix uteri with sponge tents, under such circumstances, with a view to explore the cavity with the uterine sound. If a tumor were projecting inwards, its prominency could be detected, in the same way as a stone in the bladder is found out by the surgeon. I can see no objection to this mode of procedure.

If the diagnosis be established, and a tumor ascertained, the next thing to be considered, is the treatment. I do not mean the mere palliative course, but a radical cure—if such can be affected. If the tumor belong to that class which are called polypi,—removal is the remedy. But if it be a tumor imbedded in the walls of the uterus, encroaching on the cavity of that organ, and pushing the lining membrane before it, or if it grow outwards, covered with the peritoneal membrane, this cannot be resorted to. (Perhaps I ought to say, to prevent misapprehension, that in this statement, I do not mean to include those large growths which have been so successfully removed, by ventral incisions, by Dr. Atlee and others.)

These cases are certainly very difficult to be treated. At best, as a general rule, a palliative course is the only one that promises any beneficial result. Nevertheless, the hemorrhage is sometimes permanently arrested, by a plan which prevents the growth of the tumor, and by this means relieves the congestion of the lining membrane.

It was proposed, several years ago, by Dr. Ashwell, to treat such cases by the internal exhibition of iodine, and by the inunction of iodine ointment to the neck of the womb. He did not claim that the tumor could be removed by such means, but that it could be restrained from farther development, and even sensibly diminished. And he gave the history of several cases, in which such results had been accomplished.

From our present knowledge of the nature of these tumors, it is reasonable to expect a beneficial effect from the use of iodine. A recent work on Pathological Anatomy (Jones and Sieveking) states, that "the microscopic appearances of the fibroid tumor of the uterus are not in accordance with what we should expect to find in a true fibrous structure.* * * * It appears to us, that, from the analogy they present to the genuine uterine tissue, in the unimpregnated state, we should rather class them with homologous than heterologous productions; that they should be regarded rather in a relation to the womb analogous to that of exostosis to the matrix it springs from, than of a character totally at variance with that of their nidus."

And in a note to the above, the authors go on to say, "since the above was written, a corroboration of the view expressed has been published in the Report of the Pathological Society for 1853, p. 216. Dr. Bristowe, in an elaborate paper on the subject of fibrous tumors of the uterus, concludes, from his examination of them in the impregnated and unimpregnated conditions, that all so-called fibrous tumors of the uterus, at least in their earlier stages, before degeneration has taken place in them, are essentially muscular tumors; not simply fibrous tumors, with a greater or less quantity of muscular fibre mixed up with them, but developments of true and undoubted muscular tissue."

Of the correctness of these views, there can be no doubt, I imagine. If then these tumors be nothing more than an abnormal growth of the uterine tissue, I can see no reason, why iodine should not exert over them the same influence as it does over an enlarged gland. It is no argument to assert that it has no effect over an hypertrophy of the heart—an organ somewhat similar in its structure to the womb—the causes which produce enlargement of the heart are constantly in action, and the effect is a necessary one.

At all events, I am prepared to assert that iodine will arrest, occasionally, the nutrition of the uterine tumors, and thus prevent their further development. Now I am inclined to believe that it is during the period of their growth, that the hemorrhage takes place. We rarely find that flowing continues unabated for months at a time. Previous to the flow there are pain and heaviness in the uterine region, and this is followed by the eruption of blood; and, after a short time, the pain ceases. Doubtless, the tumor at that time takes on an increased action, blood is invited to the part, the lining membrane of the uterus, under which it lies, becomes highly congested, and the hemorrhage results from such congestion—or the tumor increases inside, presses on the membrane, and actually opens the orifices of the vessels, which have a peculiar valvular arrangement as they terminate on the internal surface. When the flow has existed for a time, the congestion is relieved and the hemorrhage ceases—or the membrane accommodates itself to the pressure, and the distended orifices return to their natural condition.

Hence, it is all-important to arrest this period of growth.—This may sometimes be arrested by the timely application of a few leeches or cups, purgatives, and revulsives, when the symptoms indicate the commencement of the process. Nor are purgatives and revulsives to be neglected during any period, but the main treatment consists in the iodine. At the same time, I repeat, this as well as other means, will often disappoint us.

To illustrate its efficacy, however, I will mention two cases. The first one was unattended with hemorrhage, but the diminution of the tumor is worthy of record. About seven years ago, I was requested to see a lady, who told me that she believed that she was pregnant, as she was very much enlarged. She suffered with dragging pains in the lower part of the abdomen, and her health was impaired. She menstruated regularly. There was evidently an abdominal tumor—it was about the size of the head of a child one year old—irregular in shape, and lying to the right of the median line. She said it was sometimes larger than at others, and then it was painful—as she was thin, I could readily feel it, through the abdominal walls. I told her that she was not pregnant, and put her on the use of the iodide of potassium, and ordered iodine ointment, to be rubbed freely on the abdomen. The treatment was persevered in for several months, as she perceived a gradual diminution of the tumor. It diminished to one-half of its size, and ceased to give her any uneasiness; she has at this time no inconvenience from it, and is free from the dragging pains, and the feeling of debility, which she had when I first saw her.

The second case, was one of a lady, who suffered from recurrent menorrhagia for several years previous to my seeing her. The flow came on after a period of two or three days of pain and uneasiness in the pelvic region. These would cease in about forty-eight hours after the commencement of the flow, which lasted from one to three weeks; she would then have a period of exemption, of variable duration. Her health was much impaired. When I first saw her, she was free from hemorrhage. Examining her in the erect posture, I could detect no cause for the flow. Three weeks after, she complained of pains in the pelvis, and after two days she commenced flowing. I now had her placed on her left side, and examined by the touch—I could distinctly feel an enlargement above the anterior segment of the cervix uteri. At first I was not certain as to its nature, but became afterwards convinced, that it was a fibroid tumor. As I examined her carefully at first, and could not discover any enlargement, but as I did discover it subsequently, at the commencement of the hemorrhage, the inference was, that it increased so much in size at that time, that it came readily within reach of the finger. Two weeks after when the flow ceased, the tumor again was not to be reached. The neck of the womb was higher up than during the flow.—That these tumors do increase in size at certain times, and become painful, I have oftentimes remarked, as well in cases unattended with hemorrhage, as in those with hemorrhage.

The lady was confined to the recumbent posture during the flow, and injections of alum dissolved in water, were daily thrown in the vagina; the bowels were kept soluble. After the cessation of the discharge I applied daily, through a tubular speculum, the compound iodine ointment to the neck of the womb. Iodide of potassium was given internally, twice a day. In three weeks the flow returned and lasted two days—re-appeared in a week and lasted ten days. She now had no flow for six weeks—then it appeared and remained one week. After this the return was after an interval of twenty-four days, and it continued for a few days. From this period her menstrual periods were regular, but the flow was too abundant twice—it then became natural. I examined at the commencement of each flow, and could perceive that the tumor was gradually getting less; and it could be detected only by the most careful examination when I last performed the operation. She has continued in good health ever since.

Now, it is true, that the tumor has not totally disappeared, but its farther development has been prevented by the plan pursued.—*St. Louis Medical & Surgical Journal.*

[TO BE CONTINUED.]

